# Biofilms and Chronic Wounds: Pathogenesis and Treatment Options

**DOI:** 10.3390/jcm14217784

**Published:** 2025-11-02

**Authors:** Annabel Z. Shen, Mohamad Taha, Mahmoud Ghannoum, Stephen K. Tyring

**Affiliations:** 1 Department of Dermatology, The University of Texas McGovern Medical School, 6431 Fannin St, Houston, TX 77030, USA; 2 School of Medicine, Texas A&M University Health Science Center, Bryan, TX 77843, USA; mohamadrtaha@tamu.edu; 3 Center for Medical Mycology and Integrated Microbiome Core, Department of Dermatology, Case Western Reserve University, Cleveland, OH 44106, USA; mag3@case.edu

**Keywords:** biofilms, chronic wounds, diabetic foot ulcers, burn wounds, antimicrobial resistance, wound healing, debridement

## Abstract

**Introduction:** Chronic wounds are a growing healthcare challenge, with infections being major complications that delay healing. Biofilms are structured microbial communities encased in extracellular polymeric substances. Biofilms confer antimicrobial resistance, promote inflammation, and protect pathogens from host defenses. These mechanisms make eradication difficult with standard therapies. **Methods:** A focused literature review was conducted using PubMed (2010–2025) to examine the role of biofilms in chronic wounds, diabetic foot ulcers (DFUs), and burn injuries, as well as conventional and emerging treatment strategies. Studies are included if they addressed microbial composition, host–microbe interactions, or therapeutic outcomes in clinical or translational models. **Discussion:** Biofilms are implicated in up to 60% of chronic wounds and more than half of burn wounds. In DFUs, both bacterial and fungal biofilms contribute to chronicity and impaired healing. Conventional treatments such as debridement and antiseptics reduce surface biofilm burden but rarely achieve full eradication. Emerging approaches include quorum sensing inhibitors, bacteriophage therapy, matrix-degrading enzymes, electroceutical dressings, antifungal strategies, and nanotechnology. They show promise when integrated with standard wound care. **Conclusions:** Biofilms are central to the pathogenesis of chronic wounds, DFUs, and burns. Integrating mechanism-based antibiofilm therapies with standard care represents a key research priority to improve healing outcomes.

## 1. Introduction

Chronic wounds represent a significant and growing healthcare challenge worldwide, with infections being a major contributor to delayed healing. A key factor underlying these infections is the presence of biofilms, which are structured microbial communities encased in an extracellular polymeric substance (EPS) [1,2]. Over 90% of chronic wounds contain bacteria living within the biofilm matrix [3]. Within this matrix, microorganisms share nutrients, exchange resistance genes, and communicate through quorum sensing, enabling them to persist despite host immune responses and conventional antimicrobial therapies [4,5].

Biofilm-associated infections (BAIs) are estimated to contribute to 65–80% of all infectious diseases, as estimated by The Centers for Disease Control (CDC) and the National Institutes of Health (NIH) [6]. In the wound setting, bacterial biofilm development occurs in four stages. First, bacteria attach to surfaces using appendages such as hyphae, flagella, or receptors. Secondly, microcolonies form and release substrates that create the biofilm’s base layer. A mature biofilm then develops and includes features such as water channels and clustered cells. Bacteria and fungi are released, spreading the BAI.

The mechanism of biofilms protect pathogens by reducing metabolic activity, promoting efflux pump overexpression, and harboring highly tolerant “persister” cells that can reseed infection after treatment [7,8,9,10]. Clinically, they are often suspected in wounds with delayed healing, recurrent or refractory infection, excessive exudate, and low-grade chronic inflammation [11]. As the biofilm continues to mature, they continuously change their phenotype to survive in the lowest energy state possible [12].

Despite their impact, diagnosing BAIs remains challenging. The 2012 World Biofilm Symposium outlined key clinical signs of biofilm-infected wounds [13]. These clinical signs include (1) pale, edematous wound bed; (2), fragile granulation tissue; (3) yellow exudate; (4) necrotic tissue; (5) painful wounds; (6) putrid odor.

This guideline was updated twice, once in 2017 and again in 2019 [10,14]. The updates included (1) ineffective antibiotic results; (2) delayed healing; (3) recurring or worsening infections; (4) profuse exudate; (5) mild chronic inflammation; (6) mild swelling/redness; (7) worsening infection after stopping antibiotics; (8) effective management with debridement and antimicrobial agents.

Clinical signs may offer initial indications, but laboratory testing is highly suggested. Traditional culture and histology have limited sensitivity, while advance techniques, such as polymerase chain reaction (PCR), peptide nucleic acid fluorescence in situ hybridization (PNA-FISH), and confocal microscopy, provide higher accuracy but are not widely available in routine practice [11,15,16,17].

After the diagnosis of a BAI, treatment options depend on several factors. Newly formed biofilms are shown to be more susceptible to antibiotics and biocides due to their matrix being less developed or mature. An article by Wolcott showed that the BAIs are most susceptible to selective antibiotics within the 48 h of forming [18]. However, if the biofilm has matured, it is often resistant to biocides and antibiotic penetration [19].

Currently, debridement is seen as the standard treatment to convert a chronic wound into an acute wound. Debridement is the removal of damaged tissues or foreign objects from a wound. However, biofilms quickly spread perivascularly below the wound and reform rapidly, making it challenging for complete removal [20,21]. New studies have shown evidence that debridement, hydrotherapy, shockwave therapy, ultrasound, negative pressure with fluid instillation ability, cadexomar iodine, and biofilm-dissolving agents all have potential in removing BAIs [11]. However, there are not enough clinical trials yet to fully confirm this. It is generally accepted to give a combination therapy of debridement and topical biocides for the best wound healing [18,22,23].

Given their prevalence and role in chronicity, biofilms are now recognized as central to the pathogenesis of chronic wounds, diabetic foot ulcers (DFUs), and burn wounds. This review summarizes current understanding of biofilm formation in these contexts, highlights diagnostic and therapeutic challenges, and discusses established and emerging strategies aimed at disrupting BAIs and improving wound healing outcomes. Unlike prior published reviews, however, this manuscript differentiates bacterial and fungal biofilms in chronic wounds and highlights translational barriers to effective antibiotic therapies.

## 2. Methods

A comprehensive literature search was conducted to identify studies addressing biofilm involvement in chronic wounds, DFUs, and burns. Searches were performed in PubMed between January 2010 and October 2025 using combinations of the following keywords: biofilm, chronic wound, diabetic foot ulcer, burn wound, infection, microbiome, treatment, and antimicrobial resistance. Boolean operators (“AND,” “OR”) were used to refine results.

Inclusion criteria encompassed original research articles, clinical trials, meta-analyses, and reviews published in English that specifically discussed biofilm mechanisms, composition, or management strategies in the context of chronic wounds. Exclusion criteria included studies unrelated to wound infections, single-case reports without mechanistic data, non-English publications, and studies lacking clear biofilm focus.

The initial search yielded a broad set of studies. Articles were screened by title and abstract, and those meeting the inclusion criteria were included in the final synthesis. This review followed PRISMA-style principles to enhance methodological transparency and reproducibility.

Prevalence statistics cited in this review are drawn from the most frequently referenced systematic and narrative reviews; variations in diagnostic methods, study settings, and patient populations may contribute to differing estimates.

## 3. Chronic Wounds

In the United States, 2% of the population suffers from some type of chronic wound, presenting a substantial burden to the healthcare system [24,25]. Unlike acute wounds which typically heal within weeks, chronic wounds persist due to impaired immune function, tissue necrosis, and an environment that favors microbial colonization [7,26]. Biofilms are detected in up to 60% of chronic wounds compared to only ~6% of acute wounds [8,27].

**Figure 1:**
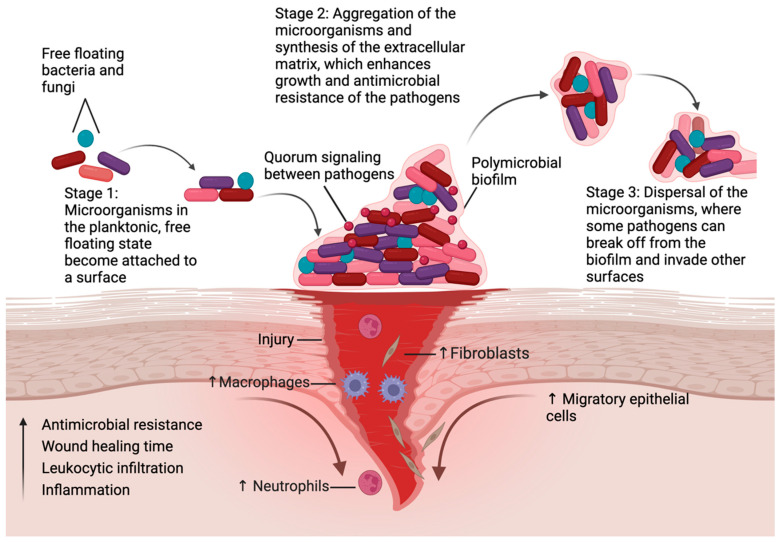
Biofilms and wound healing. Steps involved in the synthesis of biofilms, and their impact on wound healing. The first step is the attachment of microorganisms to a surface. Next, these microbes begin secreting extracellular polymeric substances that enhance their attachment and growth [4]. Finally, some organisms can break off from the original biofilm and attach to other surfaces, which leads to further colonization and damage to the host. Biofilms increase inflammation, leukocytic infiltration, antimicrobial resistance, and can significantly delay the wound healing process, resulting in the formation of chronic wounds. (Created with BioRender.com, accessed on 28 October 2025).

Normal wound healing progresses through four overlapping phases: hemostasis, inflammation, proliferation, and remodeling (Figure 1) [26,28]. In hemostasis, the blood vessels contract and the coagulation cascade seals the injured area to minimize blood loss [29]. In the second step of acute inflammation, IL-8 attracts neutrophils to the site of injury. The neutrophils engulf and kill bacteria in the area through production of reactive oxygen species and proteases. After this, proliferation begins where granulation tissue forms and the keratinocytes undergo epithelialization [30,31]. Lastly, remodeling occurs where the extracellular matrix matures, and the tissue is now stable and functional [29,32].

Disruption of the normal wound healing process leads to impaired restoration of the skin. This can involve fibrosis, keratinocyte dysfunction, and biofilm formation [16]. Biofilms perpetuate inflammation by driving leukocyte infiltration, altering cytokine profiles (e.g., IL-1β, TNF-α), and increasing protease activity [7,25,33]. These lead to degradation of the extracellular matrix [34]. The extracellular matrix components within the biofilm act as pathogen-associated molecular patterns (PAMPs). PAMPs activate immune cells through pattern recognition receptors and Toll-like receptors. This process activates the NLRP3 inflammasome and triggers the release of inflammatory cytokines [35]. Inflammatory cytokines delay healing and predisposes wounds to recurrent infections by weakening tissue tensile strength [6,36]. In addition, biofilms have a protective matrix that shields the bacteria from host immune cells and antimicrobial treatments, which leads to continued chronic inflammation [35].

Common chronic wound types include pressure injuries, venous leg ulcers, ischemic ulcers, and DFUs. Each condition presents unique risk factors and management challenges, but all share a susceptibility to biofilm formation. Among these, DFUs are the most prevalent and clinically significant, given the rising global incidence of diabetes.

The following subsection will examine DFUs in greater detail, focusing on the interplay between hyperglycemia, immune dysfunction, and biofilm development.

## 4. Diabetic Foot Ulcers

Diabetes mellitus (DM) affects over 537 million individuals worldwide, and its prevalence continues to rise [37]. A major complication is the development of DFUs, which occurs in approximately 6.3% of patients with DM globally [38]. DFUs are associated with high amputation rates, with more than 60% of non-traumatic amputations involving DFUs, and a five-year mortality of 48% [39,40,41,42].

According to the International Working Group on the Diabetic Foot, the guidelines for classifying DFUs are according to the following symptoms secondary to current or previous DM: (1) skin chapping, ulceration; (2) disruption of foot epidermis and dermis; (3) breached skin envelope; (4) exposed sterile structures; (5) and formation of full-thickness lesions [43,44].

These symptoms of DFUs are often due to impaired wound closure driven by peripheral neuropathy, vascular insufficiency, and immune dysfunction [39,45]. Due to disruption of glycemic balance, inhibition of nociception and reduction in foot skin innervation by C-fibers and autonomic nerve fibers occur. This process is called protective sensation [46]. Vascular insufficiency occurs in DM patients as the thickening of capillary basement membranes leads to decreased oxygen perfusion of tissues [47]. Decreased perfusion can potentiate ischemia and enhance the severity of wound infections due to poor delivery of oxygen and nutrients [48]. For immune dysfunction, studies have shown that persistent hyperglycemia may alter cellular functions of phagocytosis, chemotaxis, and bactericidal activity [49]. Collectively, these factors do not act in isolation but interact mechanistically to sustain a microenvironment that favors persistent biofilm formation. Impaired perfusion limits antibiotic delivery, while hyperglycemia alters immune cell metabolism. This interplay underlies the chronicity of DFUs.

Biofilms are a critical factor in the persistence of DFUs. They are found in nearly 70% of diabetic foot infections [50], most often involving Gram-negative bacteria such as *Pseudomonas aeruginosa* and *Escherichia coli* [40,51]. Biofilm communities sustain a chronic inflammatory environment dominated by ineffective neutrophil activity due to being trapped at the biofilm’s edge [52]. This results in further damage to the surrounding tissue. Quorum sensing, an intracellular communication mechanism, in organisms such as *P. aeruginosa* also promotes neutrophil recruitment and amplifies inflammation [40]. In addition, several biofilm studies on animal models of DFU have shown increased oxidative stress and reactive oxygen species promoted with BAIs [53,54,55]. This oxygen-limiting condition is due to either metabolic activity from the bacteria or oxygen deprivation by host defenses [55]. These findings suggest that biofilm-related inflammation in DFUs represent a self-perpetuating feedback loop where bacterial persistence drives neutrophil activation and tissue damage that leads to more microbial adherence.

Fungal species also play a role in DFU pathogenesis. By altering the skin barrier and interacting with bacterial species, fungi increase microbial virulence and resistance to treatment [56,57]. *Candida* species are the most frequently detected, though *Aspergillus* and *Penicillium* may also be present [56,57]. The formation of *Candida* occurs in four stages: (1) the adherence of yeast-form cells to a substrate; (2) yeast cells proliferation using hyphae and pseudohyphae; (3) maturation of the cells; (4) dispersal in surrounding environment [58].

Increased fungal diversity is correlated with delayed wound healing and hyperglycemia appears to enhance fungal virulence by upregulating enzymes such as phospholipases and proteinases [57]. Such cross-kingdom interactions likely alter local pH and nutrient gradients, stabilizing mixed biofilms that are more resistant to antifungal or antibiotic penetration. This highlights the importance of recognizing fungal biofilms not merely as co-inhabitants but as active modulators of bacterial virulence and chronicity.

Standard DFU management includes pressure offloading, vascular optimization, infection control, and debridement [59,60]. Negative pressure wound therapy, topical growth factors, and skin substitutes are often used as adjunctive measures. Antifungal therapies are not routinely incorporated into DFU protocols; however, several small clinical studies suggest benefit [61]. For example, patients receiving fluconazole in combination with standard care demonstrated faster healing compared with controls. Broader-spectrum regiments (e.g., flucytosine, itraconazole, terbinafine) have also shown improved outcomes in refractory cases [56]. Among antifungal classes, lipid formulations of amphotericin B and echinocandins appear particularly effective against *Candida* biofilms [62].

However, these findings should be interpreted with caution. Most studies are limited by small sample sizes, heterogeneous patient populations, and inconsistent methods for confirming fungal biofilm involvement. Moreover, clinical outcomes likely depend on infection chronicity, species composition, and drug penetration within mixed bacterial-fungal matrices [61]. The variable efficacy observed across trials underscores the need for standardized diagnostic criteria and controlled studies to determine when antifungal therapy provides meaningful benefit in DFU management.

## 5. Burn Wounds

Burn injuries remain a major global health concern, with infection as the leading cause of morbidity and mortality. Up to 75% of burn-related deaths are attributed to infectious complications [63]. The loss of skin barrier integrity coupled with burn-induced immunosuppression creates a highly favorable environment for microbial colonization and biofilm formation.

Biofilms form rapidly in burn wounds, often involving polymicrobial communities that exhibit higher pathogenicity, greater resistance to antibiotics, and slower healing compared with single-species biofilms [17,63]. These structures not only delay wound closure but also facilitate microbial invasion into deeper tissues, increasing the risk of sepsis and systemic infection. Biofilm prevalence in burn wounds is reported in more than 50% of cases [63].

*P. aeruginosa* is implicated in up to 77% of burn-related deaths [63]. An important factor influencing *P. aeruginosa* biofilm formation is the depletion of iron in burn environments. *P. aeruginosa* overcomes this by producing their own siderophores that scavenge iron [64]. Studies have shown that mutations of siderophores lead to weaker biofilm formations; iron acquisition is key to biofilm formation [65]. *Staphylococcus aureus* is also common and takes advantage of disrupted skin to form robust biofilms. Since *S. aureus* is commonly found on healthy skin, it colonizes quickly within the first 48 h on burn wounds [66]. *S. aureus* also induces release of leukocidins such as the Panton-Valentine leukocidin. These leukocidins trigger neutrophil death and prevent immune clearance [67]. Fungal biofilms occur less frequently but are clinically important, found in 6.3% of burn wounds [56,68].

Burn wound management parallels that of other chronic wounds but requires additional interventions due to the severity of tissue injury and the high risk of infection. Topical agents such as hydrogels, including Prontosan (polyhexamethylene biguanide with betaine), as well as experimental formulations containing moxifloxacin, chitosan, and Boswellia gum, have demonstrated significant antibiofilm activity in animal models [69,70,71]. Antibiotics and antiseptics are also important. Colistin is reserved as a last-line systemic therapy for multidrug-resistant Gram-negative infections, while acetic acid and chlorhexidine-based dressings are frequently employed for local disinfection [68,72]. In addition, biologic dressings such as fish skin xenografts have received FDA approval and shown promise in reducing infection and promoting wound healing [50]. Together, these approaches aim to reduce microbial burden, support tissue regeneration, and improve survival outcomes in burn patients.

## 6. Treatment and Strategies

**Figure 2:**
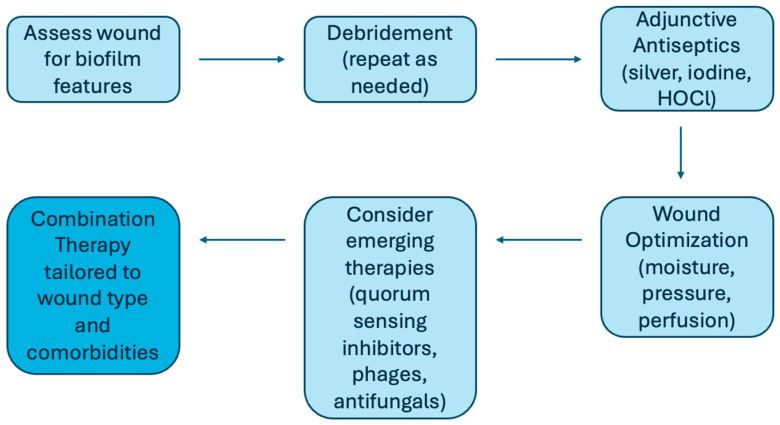
Practical clinical framework for managing BAIs. A stepwise approach to management beginning with wound assessment, followed by repeated debridement, adjunctive antiseptics, and optimization of the wound environment. Emerging therapies such as bacteriophages, quorum sensing inhibitors, matrix-degrading enzymes, electroceutical dressings, and antifungals (when indicated) may be incorporated for refractory wounds. The most effective outcomes are achieved through combination therapy tailored to wound type, microbial profile, and patient comorbidities.

Effective management of biofilm-associated wounds requires a multimodal approach that integrates conventional wound care with newer therapies designed to disrupt biofilm architecture. Given the variety of available interventions, a practical framework can help clinicians apply these strategies in a stepwise manner (Figure 2). The framework highlights how these interventions are not isolated steps, but an interdependent process between different mechanical, chemical, and molecular strategies.

Among conventional strategies, debridement remains the cornerstone, as mechanical or surgical removal of necrotic tissue reduces microbial load. However, repeated procedures are often necessary because biofilms can rapidly reform within 24 to 48 h [6,7]. Adjunctive antiseptics such as silver, iodine, and hypochlorous acid (HOCl) are used to limit regrowth, though their efficacy may vary depending on microbial strain [6,73,74]. Optimizing the wound environment is equally important, as are measures such as maintaining moisture balance, managing wound edges, and redistributing pressure through offloading, compression, or reperfusion, depending on ulcer type.

In addition to these foundational methods, a growing number of emerging and experimental therapies target the unique biology of biofilms. Quorum sensing inhibitors—including natural agents like honey, curcumin, and furanones—block microbial communication pathways essential for maturation and virulence [6,74,75]. Bacteriophage therapy selectively lyses biofilm-forming bacteria and secretes enzymes that degrade the extracellular matrix, with early studies suggesting synergy with antibiotics [6]. Matrix-degrading enzymes such as DNase I, dispersin B, and amylases enhance antibiotic penetration, while antimicrobial peptides and surfactants—including cathelicidin /LL-37, defensins, manuka honey, and rhamnolipids—interfere with microbial membranes and adhesion [76,77]. Electroceutical dressings generate microcurrents that disrupt quorum sensing and bacterial resistance, with early clinical use demonstrating safety [6,60]. Other adjunctive approaches such as ultrasound therapy, maggot debridement, and HOCl are also being investigated for strain-specific antibiofilm effects [6,59].

Another emerging therapy for BAIs is nanotechnology. Oxide nanoparticles, such as AgO, CuO, and ZnO, exhibit antimicrobial properties that can penetrate the biofilm matrix and directly target the bacteria within [78]. ZnO nanoparticles have demonstrated effective biofilm inhibition in food-borne pathogens like *S. aureus*, *S. enterica*, and *E. coli* through modification of surface adhesion, hydrophobicity, and reactive oxygen species generation [79]. CuO and AgO nanoparticles show antibiofilm activity, especially against MRSA and *E. coli*, by disrupting the bacterial envelope and generating ROS to cause cell death [80,81].

Despite promising preclinical data, clinical trial evidence remains limited, and translation into standard care is slow [78]. The most effective outcomes are likely to come from combination strategies that pair established treatments such as debridement with novel antibiofilm agents [69,82]. A summary of current treatment strategies and their mechanisms of action is provided in Table 1.

## 7. Cross-Wound Comparisons and Shared Mechanisms

Despite differences in etiology, chronic wounds, DFUs, and burns share core biofilm-mediated mechanisms that delay healing. Across all wound types, biofilms enable immune evasion, chronic low-grade inflammation, and antimicrobial resistance through quorum sensing, altered cytokine signaling, and matrix-mediated protection.

However, each wound presents distinct microenvironmental challenges. DFUs are characterized by hyperglycemia, neuropathy, and impaired perfusion. These characteristics create hypoxic, nutrient-rich areas that promote bacterial-fungal synergy. Studies show that bacterial cells can bind directly to *Candida* hyphal cells in the biofilm through either fungal adhesion or wall proteins [88]. In contrast, burn wounds feature acute and thermal immunosuppression and disrupted epithelial barriers favoring early colonization by *Pseudomonas* and *Staphylococcus*. Chronic venous and pressure ulcers develop under conditions of sustained ischemia and fibrin deposition that favor mixed aerobic-anaerobic communities.

Recognizing these shared and divergent patterns provides an opportunity to develop tailored antibiofilm interventions—linking wound specific physiology with mechanism-based treatment design.

## 8. Conclusions

Biofilms are a central barrier to healing in chronic wounds, diabetic foot ulcers, and burn injuries [16,40,51]. By creating a protected microbial niche, they perpetuate inflammation, confer antimicrobial resistance, and contribute to recurrent or refractory infections. Conventional therapies—particularly repeated debridement and antiseptic application—remain essential, but are rarely sufficient to eradicate biofilms completely [6,7].

Moving forward, progress will depend on bridging the gap between research and clinical application. Standardized biofilm models are needed to allow reproducible testing of therapies, such as frameworks like BRIEF [89]. Further work is required to clarify the regulatory and signaling pathways that control biofilm formation and dispersal. A deeper understanding of fungal–bacterial interactions and host–microbe dynamics will also be critical for designing targeted, combination treatments.

Future research should prioritize translational studies and clinical trials that evaluate multimodal antibiofilm approaches. This includes quorum sensing inhibitors, bacteriophage therapy, electroceutical dressings, matrix-degrading enzymes, and nanotechnology. These novel approaches hold promise, particularly when used in combination with standard care [69,82].

## Figures and Tables

**Table 1:** Summary of treatments used in the management of biofilms along with their mechanism of action [6,11,62,69,78,79,80,81,82,83,84,85,86,87].

**Treatment**	**Examples**	**Action**	**Evidence Level**	**Translational Readiness**
Debridement	Physical debridement, ultrasound therapy	Removes necrotic tissue and biofilm from wound surface	Clinical	Widely established in practice
Antiseptics	Silver, iodine	Antimicrobials, some evidence of reducing or preventing biofilm formation	Clinical	Routinely used; strain-specific efficacy
Hydrogels	Prontosan, Carrasyn, Curagel, Nu-Gel, Purilon, Restore, SAF-gel, XCell	Anti-biofilm activity, particularly against *S. aureus* and *P. aeruginosa*	Clinical	FDA-approved wound dressings
Amphotericin B	Liposomal AMB and AMB lipid complex	Binds to ergosterol, leading to formation of pores in the fungal cell membrane, ion leakage and cell death	Clinical	Moderate adoption; limited DFU data
Echinocandins	Caspofungin, micafungin	Inhibit beta-(1,3)-D-glucan synthase, an enzyme required for fungal cell wall synthesis. This results in decreased cell wall stability, which dysregulates osmotic pressure, leading to cell lysis	Clinical	Moderate adoption; limited DFU data
D-Amino acids	d-Leu, d-Met, d-Trp, d-Tyr	Enhances antibiotic activity against biofilms, particularly *S. aureus* and *P. aeruginosa*	Preclinical	In vitro and animal data
Light	Antimicrobial blue light	Anti-biofilm activity against *Acinetobacter baumannii* and *P. aeruginosa*	Preclinical/Early Clinical	Pilot trials; emerging adjunctive therapy
Bacteriophages	-	Target bacteria with high specificity, secrete enzymes that disrupt biofilm matrix	Preclinical	Expanding clinical trials
Small molecules	Mannosidase, trypsin enzymes, platensimycin, salicylidene acylhydrazide	Various mechanisms of anti-biofilm activity, target matrix proteins, microbial membranes and virulence factors	Preclinical	Conceptual; in vitro efficacy
Quorum sensing inhibitors	Azithromycin, bergamottin, ellagic acid, quercetin, usnic acid	Inhibit biofilm formation and reduce bacterial virulence	Preclinical	Natural compounds under investigation
Matrix degrading enzymes	DNAase I, Dispersin B, a-amylase	Degrade biofilm structures which increases antibiotic penetration	Preclinical	Limited human data
Surfactants	Sodium dodecyl sulfate, rhamnolipids, poloxamer-188	Inhibit microbial adhesion and interfere with EPS matrix formation	Preclinical	Potential wound care additives
Antimicrobial peptides	Nisin A, human cathelicidin/LL-37, human beta-defensin 3, manuka honey	Antimicrobial activity, bind to negatively charged molecules on the microbial membrane	Preclinical/Early Clinical	Natural peptides under early evaluation
Nanoparticles	CuO, ZnO, AgO	Modification of surface adhesion and generation of reactive oxygen species generation	Preclinical	Proof-of-concept; active materials research
